# ADHD and Covid-19: current roadblocks and future opportunities

**DOI:** 10.1017/ipm.2020.53

**Published:** 2020-05-21

**Authors:** J. McGrath

**Affiliations:** Department of Psychiatry, Trinity Centre for Health Sciences, St. James’s Hospital, Dublin 8, Ireland

**Keywords:** ADHD, attention deficit hyperactivity disorder, child and adolescent psychiatry, coronavirus, Covid-19, Ireland, telemedicine, telemental health, telepsychiatry

## Abstract

Attention deficit hyperactivity disorder (ADHD) is the commonest disorder presenting to Child and Adolescent Mental Health Services in Ireland. This article considers the impact of the Covid-19 pandemic on the provision of mental health services for young people with ADHD with specific reference to the difficulties that have been experienced in ADMiRE, a specialist ADHD service in Dublin, since the outbreak of Covid-19 in Ireland. Current guidelines and alternative ways of ensuring adequate service provision are discussed. Restrictions to mitigate the spread of Covid-19 are likely to continue for many months, and child and adolescent mental health services need to find new ways to provide a sustainable service to young people in Ireland. There is a growing evidence base for the use of telepsychiatry in the assessment and management of ADHD. Factors that should be considered when developing a telepsychiatry service for children and adolescents with ADHD are highlighted.

Attention deficit hyperactivity disorder (ADHD) is a neurodevelopmental disorder characterised by inattention, hyperactivity and impulsivity that is developmentally inappropriate and causes significant functional impairment (Thapar & Cooper, 2016). It is one of the commonest childhood psychiatric conditions with a worldwide prevalence estimate of 5.3% (Polanczyk *et al.* 2007). In Ireland, ADHD is the commonest presentation to Child and Adolescent Mental Health services (CAMHS); approximately one-third of children in Irish CAMHS have a diagnosis of ADHD (Health Service Executive (HSE), 2014).

ADHD shows high concurrent comorbidity with other neurodevelopmental disorders including autism spectrum disorder (ASD), specific learning or motor disorders (e.g. reading disability, developmental coordination disorder), intellectual disability and tic disorders (Jensen & Steinhausen, 2015) and with behavioural problems including oppositional defiant and conduct disorders (Taylor *et al.* 1996; Jensen & Steinhausen, 2015). A diagnosis of ADHD is associated with low academic attainment and premature cessation of education (Loe & Feldman, 2007), serious antisocial behaviour, involvement with the police and substance misuse in adolescence (Langley *et al.* 2010; Mohr-Jensen & Steinhausen, 2016). Recent large pharmacoepidemiological studies have demonstrated that effective pharmacological treatment of ADHD significantly reduces the risk of these negative outcomes (Chang *et al.* 2019).

Despite the high prevalence and significant impact of this disorder, children referred with symptoms of ADHD often experience delays before assessment and intervention in CAMHS, as teams struggle to keep up with the growing demand for emergency assessments. Early intervention can make significant positive differences to outcomes for children and adolescents with ADHD (Sonuga-Barke & Halperin, 2010).

In September 2018, ADMiRE, a tertiary-level specialist ADHD pathway, was introduced in South Dublin Linn Dara CAMHS in order to provide early access to evidence-based assessment, diagnosis and intervention for children who are referred with primary presenting difficulties suggestive of ADHD. ADMiRE has implemented an evidence-based standardised model of care for assessment and intervention for ADHD that is based on the Dundee Clinical Care Pathway (Coghill & Seth, 2015). This Clinical Care Pathway has demonstrated that a careful standardised titration of medication including guideline-based management of adverse effects makes a significant difference in terms of sustained improvement of measured ADHD outcomes when compared to a less-structured approach (Coghill & Seth, 2015). In line with the Dundee Clinical Care Pathway and the National Institute for Health and Care Excellence (NICE) guidelines (National Institute for Health and Care Excellence, 2018), the model of care in ADMiRE involves structured assessment and observation, and interventions offered include psychoeducation, pharmacotherapy and ADHD-specific groups including a parenting programme.

## Impact of Covid-19 on ADHD service provision, international guidelines and alternative ways of working

As confirmed cases of Covid-19 began to rise in Ireland in March 2020, there was a corresponding reduction in face-to-face appointments nationally across the health service. Paediatric outpatient departments closed and the majority of GPs stopped seeing patients in person. Child mental health services replaced face-to-face appointments with telephone or video consultation, and in ADMiRE, there were very few in-person consultations in late March and early April 2020. Initially, it was very difficult to provide an adequate ADHD service; however, during April and early May 2020, innovative and alternative methods of service delivery have been developed. The publication of guidelines on assessment and management of ADHD during Covid-19 by both the European ADHD Guidelines Group (EAGG) (Cortese *et al.* 2020*a*) and also by the Canadian ADHD Resource Alliance (CADDRA, 2020) has been helpful in confirming the approaches that should be considered when providing an ADHD service. The difficulties faced in ADMiRE and the alternative approaches that have been developed are outlined in the following section.

### Postponement of new patient assessments

In the first 2 weeks following the implementation of Covid-19 restrictions, new patient assessments were carried out via telephone as videoconferencing facilities were not available. As it was not possible to carry out adequate observation of the young person, it was not feasible to complete these assessments, and diagnosis and interventions were therefore delayed. Parents reported their frustration at this, and as a result, all new patient assessments were temporarily postponed.

EAGG guidelines (Cortese *et al.* 2020*a*) recommend that all appointments including initial assessments should continue, but they should take place remotely using telephone or videoconferencing in line with telepsychiatry recommendations (AACAP, 2017). Videoconferencing facilities are now available in ADMiRE, and this has facilitated adequate new patient assessment.

### Lack of school feedback

Closure of schools has posed significant difficulties for optimization of pharmacological intervention in ADMiRE. Typically, a baseline school report and quantitative rating scales are sought from schools prior to initiation of medication, and teacher feedback is requested regularly during titration of medication. School teachers have continued to provide baseline information about students undergoing ADHD assessment, which has been valuable in initial assessment; however, without feedback during medication titration, it is very difficult to determine the effectiveness of medication. Parental report is likely to be helpful. At present, many parents are currently home-schooling their children and have a unique opportunity to comment on the effectiveness and impact of medication on their child’s academic and social functioning; nonetheless, the lack of academic structure and routine in the current climate makes it challenging to optimise ADHD medication.

### Delays in initiation of medication

Prior to the outbreak of Covid-19, a number of young people had been diagnosed with ADHD in ADMiRE and were waiting to start on medication. In line with NICE guidance (National Institute for Health and Care Excellence, 2018), the protocol for pre-medication workup in ADMiRE includes baseline rating scales of ADHD severity and medication side effects, cardiovascular screening and a physical cardiovascular examination completed by the young person’s GP. As the Covid-19 outbreak intensified, GPs were only offering face-to-face appointments in emergency situations and did not have the capacity to complete the cardiovascular examination for these young people. For those without an adequate baseline cardiovascular workup, initiation of medication was delayed.

The new EAGG guidelines suggest that when indicated, medication for ADHD should be commenced during the pandemic as symptoms of untreated ADHD are likely to negatively impact on the family dynamic and may increase health risks for COVID-19 infection for patients, their family and others (Cortese *et al.* 2020*a*). An addendum to these guidelines provides additional advice in relation to starting ADHD medication during the Covid-19 pandemic (Cortese *et al.* 2020*b*). It is recommended that it is appropriate to start ADHD medication without a baseline cardiac auscultation if three specific conditions are satisfied. Firstly, a cardiovascular risk screen is required which includes assessment of risk symptoms in the young person (for details please see Cortese *et al.* 2020*b*); secondly, the young person with ADHD must not have a family history of early (<40 years) sudden death in a first-degree relative suggesting cardiac disease and finally, baseline blood pressure and heart rate monitoring must be carried out before initiation (Cortese *et al.* 2020*b*). If practical to do so, baseline monitoring may be carried out remotely and should be recorded on three separate occasions (details in appendix in Cortese *et al.* 2020*a*). If the first or second condition is not satisfied, referral to a cardiologist is recommended. If baseline monitoring is not possible remotely, the clinician needs to weigh up the risks and benefits of face-to-face consultation to complete physical assessment (Cortese *et al.* 2020*b*).

The Canadian ADHD Resource Alliance (CADDRA) have also provided guidelines for management of ADHD during the pandemic and suggest that a physical examination should be conducted with the appropriate use of personal protective equipment (PPE) prior to starting ADHD medication (CADDRA, 2020). CADDRA also suggests that clinicians could consider whether there is a record of recent (<6 months) physical examination including blood pressure, heart rate, weight and height (CADDRA, 2020).

### Delays in titration and optimisation of ADHD medication

In relation to pharmacotherapy, of the 210 young people attending ADMiRE, 177 (84%) are prescribed ADHD medication. The majority (85%) are on a psychostimulant [Methylphenidate (*n* = 131) or Lisdexamphetamine (*n* = 20)], 12% are on Atomoxetine and 5% are on Guanfacine (both as monotherapy and stimulant augmentation). 37% are also prescribed Melatonin. NICE guidelines recommend that for young people on ADHD medication, blood pressure and heart rate are monitored before and after each dose change and every 6 months, height is monitored every 6 months and weight every 3 months (for <10 years) or 6 months (>10 years) (National Institute for Health and Care Excellence, 2018). In addition, response to medication (ADHD severity ratings) and side-effect ratings should be recorded.

With the reduction in face-to-face appointments, it became extremely difficult for clinicians in ADMiRE to arrange adequate physical monitoring. Young people were invited to attend ADMiRE for physical review if required; however, the vast majority declined, as families were anxious to remain at home. GP referral was not feasible, and local pharmacies were not providing blood pressure measurement. As a result, medication titrations were paused for many young people at a time when an optimised dose of ADHD medication may have been particularly beneficial for the young person and their family.

The new EAGG guidelines recommend that routine cardiovascular monitoring for those without any cardiovascular risk factors may be postponed until clinics re-open, but they suggest that home-based monitoring may be possible for families who have access to a sphygmomanometer (Cortese *et al.* 2020*a*). The guidelines stress the need for clinicians to educate families about the urgent need to report any emerging cardiovascular symptoms such as palpitations, shortness of breath or chest pain.

There has been limited practical guidance to date about physical monitoring of those undergoing medication titration. A variety of options have been considered and trialled in ADMiRE. If families have a sphygmomanometer, smartphone and weighing scales, home physical monitoring is possible. The majority of families do not have access to blood pressure monitoring equipment however. Some local pharmacies will record blood pressure and heart rate, but many are not providing this service during the pandemic. GPs are now offering more face-to-face appointments, and some families have opted to attend their GP for cardiovascular monitoring. In ADMiRE, a dedicated physical monitoring clinic has been set up so young people attend the service at set times, similar to the current practices in GP surgeries. In general, one of these options will be feasible for a family, and clinicians in ADMiRE are now continuing medication titration with adequate physical monitoring.

### Prescription of controlled drugs

When Covid-19 restrictions were introduced in Ireland in March 2020, ADMiRE clinicians received a surge of requests for prescriptions for stimulant medication as families were concerned about future supply and accessibility. Stimulant medications are classified as Schedule 2 controlled drugs in Ireland and are subject to the Misuse of Drugs Acts 1977 to 2016 and the Misuse of Drugs Regulations 2017.

Controlled drug prescriptions can be directly dispensed from a pharmacy if the family are paying for the medication, or if the young person has a Long-Term Illness book. The majority of young people attending ADMiRE however have a Medical Card and claim their medication under the General Medical Services (GMS) Scheme. Under this scheme, the GP is required to transcribe the prescription onto a GMS script, which is then brought to the pharmacy by the family. There were two difficulties with this process especially in the early days of the Covid-19 pandemic. Firstly, it involved an excessive number of journeys for families between the mental health service, GP and pharmacy, which exposed them to unnecessary risk. Secondly, GP services were overwhelmed and many families reported that they were unable to access their GMS prescription. At this time, many pharmacists adopted a flexible approach, whereby they dispensed non-GMS prescriptions at no charge in order to assist patients who were unable to get the GMS prescription from their GP. However for controlled drug prescriptions for stimulants, pharmacists were unwilling to dispense without the GMS script leaving many patients from ADMiRE without medication. By early April 2020, GPs were accepting email prescriptions from ADMiRE clinicians for GMS patients and were emailing the GMS scripts directly to the patient’s pharmacy necessitating only one journey for the parent to collect medication.

### Concern about guidelines for use of PPE

Initial HSE guidelines for use of PPE in ADMiRE and community CAMHS appeared to differ from the practice that was being followed by GPs and by clinicians in paediatric Emergency Departments. These initial guidelines recommended a phone risk assessment, with face-to-face appointments only to be offered to young people if the family were asymptomatic. For those who were asymptomatic, it was suggested that no PPE was required, that clinicians and families should wash their hands at the start and end of review, sit 2 metres or more from each other and that face-to-face appointments could last for 15 minutes, followed by a short break before continuing for a further 15 minutes.

Clinicians in ADMiRE became increasingly uncomfortable about face-to-face contact using these guidelines as emerging epidemiological data from March and early April 2020 suggested that the majority of children with Covid 19 infection may be asymptomatic (Dong *et al.* 2020) and that the disease is far more contagious than initially thought with a reproductive ratio (R0) of 2.76–3.25 (Liu *et al.* 2020). In recognition of this, clinicians in paediatric Emergency Departments in Ireland began working under the assumption that all children presenting to the ED had Covid-19 infection and took appropriate precautions with PPE. Guidelines for use of PPE for face-to-face appointments in ADMiRE were altered in recognition of the emerging data, and adequate PPE has been made available for clinicians carrying out physical monitoring in young people with ADHD.

### Reduction in referral rate

Referral rate has dropped by approximately 80% since the emergence of Covid-19 in Ireland. This is not surprising; teachers are not contacting parents with ADHD-related concerns, and many parents are avoiding non-essential GP visits. When restrictions ease, it is probable that there will be a surge of referrals for a variety of mental health concerns including ADHD.

### Difficulties reported by families

A number of themes emerged during telephone consultations in ADMiRE during the first 4 weeks following the implementation of Covid-19 restrictions.

In almost all cases, parents/caregivers reported an improvement in the behaviour and mood of the young person since schools had closed. Young people who were optimised on ADHD medication tended to remain on the medication and were doing reasonably well. In line with the EAGG guidance, ADMiRE clinicians altered the timing/formulation of stimulant medication in some cases to gain optimal ADHD symptom control if young person’s routine had changed significantly during the pandemic. The guidelines also suggest that ‘drug holidays’ may not be beneficial during the current restrictions and weekend use of medication should be discussed with families (Cortese *et al.* 2020*a*).

Young people with ADHD and comorbid ASD were reported to be struggling with the lack of structure and routine, and this was reflected in an increase in irritability, oppositionality and behaviours that challenge. EAGG guidance cautions against increasing stimulants or introducing antipsychotic medication to manage crises/stress related to confinement (Cortese *et al.* 2020*a*).

Sleep patterns had deteriorated for the majority of families, with sleep onset times pushed forward by approximately 3 hours. Effectiveness of Melatonin had reduced; this was felt to be related to poor sleep hygiene, significant increases in screen time and inappropriate timing of administration. The EAGG guidelines acknowledge that initial insomnia may be exacerbated by stimulant medication but recommend that clinicians should consider the impact of stress and the disruption of daily routines before adding or increasing Melatonin above 5–6 mg nocte (Cortese *et al.* 2020*a*). Interestingly, a number of adolescents with ADHD and conduct disorder were reported to have improved behaviourally and were respecting restrictions; this was attributed to their concern over vulnerable family members.

## Looking to the future – the role of telepsychiatry

A recent correspondence article in the Lancet highlights the needs of those with mental health disorders during the Covid-19 pandemic (Yao *et al.* 2020), and it is vital that mental health services return to an adequate level of functioning for young people in Ireland as soon as possible. While it is not practical or economically feasible to continue with the current Covid-19 restrictions indefinitely, it is likely that physical distancing and other mitigating measures will continue in Ireland until an effective vaccine is available for the population. It is therefore necessary to consider how to optimise alternative ways of working that will permit sustainable, effective assessment and intervention for young people with ADHD over the coming months.

Telemedicine refers to the use of technology such as videoconferencing to deliver healthcare remotely, without an in-person visit. When psychiatric assessment and intervention or general mental health services are provided by telemedicine, the terms telepsychiatry and telemental health are often used (Turvey *et al.* 2013; Shore *et al.* 2018). Telepsychiatry has been used in children and adolescents in a wide range of conditions [see (Gloff *et al.* 2015) for detailed review] and in settings including home (Comer *et al.* 2015), primary care (Goldstein & Myers, 2014) and in schools (Stephan *et al.* 2016). A growing evidence base suggests that it is a practical, acceptable and effective method of providing psychiatric assessment and interventions including pharmacotherapy, behaviour therapy and psychotherapy (Myers *et al.* 2008; Gloff *et al.* 2015; AACAP, 2017).

ADHD is a disorder that is perhaps particularly suited to telepsychiatry; it is highly prevalent and impairing, there are well-established guidelines for the assessment and management of ADHD (Coghill & Seth, 2015; National Institute for Health and Care Excellence, 2018) and there are very effective medications in the form of stimulant and non-stimulant drugs (Cortese *et al.* 2018). A recent systematic review investigating the effectiveness of telepsychiatry in the treatment of ADHD suggests that it may be ‘a viable method to provide assessment and evidence-based pharmacologic treatment for children with ADHD’ (Spencer *et al.* 2020). There is however a limited literature in this area; the authors of this systematic review found only 11 suitable articles, which were published from three trials of telemedicine in ADHD. Moreover, these articles focus primarily on the effectiveness of telemedicine when compared with primary care treatment of ADHD in rural or underserved communities. One study however has demonstrated that it is possible to use telepsychiatry to perform high-quality ADHD assessment that adheres to the American Academy of Paediatrics guidelines for evaluation of ADHD (Nelson *et al.* 2012). EAGG guidelines stress the importance of behavioural parent training strategies in ADHD, and while face-to-face training is not currently feasible, parents should be encouraged to use evidence-based self-help (Daley & O’Brien, 2013; Dose *et al.* 2017; Katzmann *et al.* 2017). It will be important for future research to determine the effectiveness of telepsychiatry compared with face-to-face assessment and intervention for ADHD in specialist child mental health services.

## Considerations when providing a telepsychiatry service for children and adolescents

The American Psychiatric Association (APA) and the American Telemedicine Association (ATA) have published a guide on best practices in clinical videoconferencing in mental health (Shore *et al.* 2018). While telepsychiatry offers an opportunity to provide assessment and intervention for ADHD via videoconferencing in the young person’s home during the Covid-19 pandemic, there are a number of factors that should be considered.

### Technological considerations

The healthcare organisation must ensure that the selected videoconferencing application has appropriate verification and security. The quality of the videoconferencing is extremely important; high bandwidth and resolution allow the clinician to engage fluidly with the young person and caregiver and facilitates development of rapport and a therapeutic relationship. It also allows the clinician to observe subtleties in the child’s facial expressions, speech and behaviour. Camera location and angle are also important; however, these factors cannot always be easily modified in a patient’s home using their existing technology. The clinician should have a backup plan in place (e.g. phone access) if technical difficulties cause a disruption of the videoconferencing session.

### Setting

During a telepsychiatry session, both locations are considered the patient examination room, and it is important to consider the therapeutic space and level of privacy that is available for a young person and their caregiver in their home as this will vary significantly from family to family. In the case where the videoconferencing is taking place in professionally unsupervised settings such as the patient’s home, the parent/caregiver needs to be able to set up the videoconferencing system and select a suitable and private space.

### Establishing a therapeutic alliance

The ability of the parent/caregiver to engage in the videoconference should be considered. It may be particularly difficult for them to encourage a young person with ADHD to attend a videoconference in their home environment. For adolescents, a quiet room with minimal distractions may be most appropriate, but for younger children, a room with some toys and activities may increase interest in the session and facilitate engagement with the parent and clinician. Engagement with adolescents may be increased through exploration of relevant and appropriate material on YouTube or other sites. Younger children may enjoy using online drawing apps and can be encouraged to tell stories while drawing.

### Physical examination

As discussed above, physical examination including blood pressure, heart rate, weight and height is routinely required during pharmacotherapy for ADHD (National Institute for Health and Care Excellence, 2018). During the Covid-19 restrictions, it has been difficult to ensure adequate cardiovascular monitoring without face-to-face reviews.

Going forward, it may be possible to use technology that until now has not been widely used in physical monitoring for young people on ADHD medication. Heart rate monitors have been included as standard in smartphones and fitness watches for a number of years. Heart rate can be accurately monitored using contact photoplethysmography (PPG) (contact of fingertip with built-in camera) or non-contact PPG, whereby the camera is used to measure changes in the volume of the surface of the body (e.g. Li *et al.* 2019; Maestre-Rendon *et al.* 2020). Most families have access to a smartphone or fitness watch, therefore home-monitoring of heart rate is currently a feasible option.

In relation to blood pressure monitoring, there are a multitude of smartphone apps that claim to accurately record blood pressure; however, many require external equipment, and the accuracy of these has been low (Jamaladin *et al.* 2018). A recent study however has reported that blood pressure can accurately be measured using a novel method called transdermal optical imaging. This technology uses a smartphone camera to capture images of imperceptible facial blood flow and uses machine learning to determine blood pressure from these images (Luo *et al.* 2019). This technology requires further validation but may facilitate home blood pressure monitoring in the near future

### Telepsychiatry prescribing

If the young person with ADHD has a Long-Term Illness book or if the family are paying directly for their medication, the prescription can be emailed directly to the pharmacy using a secure email address, and if the young person has a Medical Card, the prescription can be emailed to the GP practice where it is transcribed to a GMS script and emailed on to the pharmacy. These processes minimise face-to-face contact for both healthcare workers and families.

## Conclusion

The Covid-19 pandemic has had a significant impact on the provision of mental health services for children and adolescents with ADHD in Ireland. With ongoing Covid-19 restrictions anticipated over the coming months, there is an urgent need to find effective new ways of working. There is a growing evidence base for telepsychiatry in assessing and treating young people with ADHD; this is a practical approach that could be considered by CAMHS nationally.
